# Intertwined pathways for Argonaute-mediated microRNA biogenesis in *Drosophila*

**DOI:** 10.1093/nar/gkt1038

**Published:** 2013-11-12

**Authors:** Jr-Shiuan Yang, Peter Smibert, Jakub O. Westholm, David Jee, Thomas Maurin, Eric C. Lai

**Affiliations:** ^1^Department of Developmental Biology, Sloan-Kettering Institute, 1275 York Ave, Box 252, New York, NY 10065, USA and ^2^Molecular Biology Program, Weill Graduate School of Medical Sciences, Cornell University, New York, NY 10065, USA

## Abstract

Although Dicer is essential for general microRNA (miRNA) biogenesis, vertebrate *mir-451* is Dicer independent. Instead, its short pre-miRNA hairpin is ‘sliced’ by Ago2, then 3′-resected into mature miRNAs. Here, we show that *Drosophila* cells and animals generate functional small RNAs from *mir-451*-type precursors. However, their bulk maturation arrests as Ago-cleaved pre-miRNAs, which mostly associate with the RNAi effector AGO2. Routing of *pre-mir-451* hairpins to the miRNA effector AGO1 was inhibited by Dicer-1 and its partner Loqs. Loss of these miRNA factors promoted association of *pre-mir-451* with AGO1, which sliced them and permitted maturation into ∼23–26 nt products. The difference was due to the 3′ modification of single-stranded species in AGO2 by Hen1 methyltransferase, whose depletion permitted 3′ trimming of Ago-cleaved pre-miRNAs in AGO2. Surprisingly, Nibbler, a 3′–5′ exoribonuclease that trims ‘long’ mature miRNAs in AGO1, antagonized miR-451 processing. We used an *in vitro* reconstitution assay to identify a soluble, EDTA-sensitive activity that resects sliced pre-miRNAs in AGO1 complexes. Finally, we use deep sequencing to show that depletion of *dicer-1* increases the diversity of small RNAs in AGO1, including some candidate *mir-451*-like loci. Altogether, we document unexpected aspects of miRNA biogenesis and Ago sorting, and provide insights into maturation of Argonaute-cleaved miRNA substrates.

## INTRODUCTION

microRNAs (miRNAs) are an abundant and diverse class of ∼22 nucleotide (nt) regulatory RNAs that collectively play essential roles during development and physiology (1,2). Generally speaking, miRNAs are precisely defined small RNAs that derive from endogenous inverted repeat transcripts (3). In animals, most miRNAs are generated via a canonical mechanism involving compartmental, sequential processing (4). Primary miRNA transcripts are first cleaved near the base of the hairpin by the nuclear Drosha RNase III enzyme to generate a ∼55–70 nt pre-miRNA bearing a ∼2 nt 3′ overhang. The pre-miRNA 3′ overhang, which is characteristic of RNase III cleavage, is recognized by Exportin-5, which transports it to the cytoplasm. There, the hairpin is cleaved near the terminal loop by the Dicer RNase III enzyme to yield a miRNA/miRNA* (star) duplex that is loaded into an appropriate Argonaute effector protein and matured into a single-stranded regulatory complex (5).

Since the recognition of the core canonical pathway for miRNA biogenesis, several non-canonical strategies have emerged. These include a variety of Drosha-independent mechanisms, which can exploit the splicing machinery, snoRNA pathway, RNA exosome, RNase Z or the Integrator complex to define pre-miRNA substrates (6). Conversely, a Dicer-independent mechanism for miRNA biogenesis was ascribed to the conserved vertebrate locus *mir-451* (7). Cleavage of its primary transcript by Drosha generates a 42 nt pre-miRNA whose duplex is too short to serve as a Dicer substrate. Instead, direct loading of *pre-mir-451* into the Slicer enzyme Ago2 results in cleavage of its 3′ arm to generate a 30 nt Ago-cleaved pre-miRNA (ac-pre-miRNA), whose 3′ terminus is resected to yield mature ∼23 nt miR-451 (8–11). Since short hairpins analogous to *mir-451* have not yet been identified in well-studied models such as *Caenorhabditis elegans* and *Drosophila melanogaster*, the capacity for direct Ago-mediated miRNA biogenesis in invertebrates is not known.

Pathways for small RNA biogenesis diversified in arthropods by the emergence of two Dicers; vertebrate genomes encode only one. For example in *Drosophila*, Dcr-1 is specialized for miRNA biogenesis, whereas Dcr-2 is dedicated to siRNA biogenesis (12). This is accompanied by a division in labor for the two *Drosophila* Ago proteins: both have Slicer activity, but AGO1 mediates miRNA function while AGO2 mediates siRNA function (13). However, dicing and Ago loading are not directly coupled. Instead, small RNA duplexes are inspected for the relative thermodynamic energy of their termini (14–16), their 5′ nucleotide identity and their central structure, to determine which Ago complex they are sorted to and which strand is preferentially selected as the guide (17–21). In general, AGO2 prefers duplexes that are highly paired, especially at positions 9–10 of the 5′ end of the strand selected as the guide, and prefers 5′ C, whereas AGO1 prefers centrally unpaired duplex nucleotides, is partial to 5′ U, and selects its guide strand predominantly on the thermodynamically unstable duplex end (5).

The independent assessment of duplexes for Ago sorting means that certain Dcr-1 products, including specific mature miRNAs and many miRNA* strands, are preferentially sorted to AGO2 (19–22), while some Dcr-2 products can productively load AGO1 (23,24). In particular, loss of R2D2, a double-stranded RNA binding domain (dsRBD) partner of Dcr-2 that determines siRNA strand selection, or loss of AGO2 itself, both result in substantial redirection of endo-siRNA duplexes to AGO1 (25,26). A further twist is the recent finding that invertebrate and vertebrate Ago proteins are capable of loading some endogenous single-stranded RNAs, including the terminal loops of select miRNA hairpins (27,28).

In this study, we examined the capacity of *Drosophila* cells and animals to mature miRNA hairpins via a Slicer-mediated mechanism. We show that *mir-451*-class hairpins can generate functional miRNAs with regulatory activity, but the majority of products arrest at the 30 nt ac-pre-miRNA stage. This was due to preferential loading of the well-paired *pre-mir-451*-class hairpins into AGO2; hairpins that loaded AGO1 were successfully resected into mature miRNAs. Surprisingly, although removal of RNAi factors results in substantial re-sorting of endo-siRNAs into AGO1 (25), this condition did not enhance accumulation of miR-451 into AGO1. Instead, the maturation of miR-451 was strongly stimulated by loss of Dicer-1, likely due to loss of the competing canonical miRNA pathway. As with mature endo-siRNAs (5,29), the 3′ ends of *pre-mir-451* hairpins were modified in AGO2, and depletion of Hen1 permitted resection of *ac-pre-miRNAs* in AGO2. Notably, the miR-451 resectase does not correspond to the 3′–5′ exoribonuclease Nibbler, which was recently shown to trim the 3′ ends of *Drosophila* miRNAs in AGO1 (30,31). In fact, loss of Nibbler actually stimulated the accumulation of mature resected miR-451. We used an *in vitro* reconstitution assay to identify an EDTA-sensitive activity that mediates 3′ trimming of *ac-pre-mir-451* molecules, which sets the stage for biochemical purification of the underlying activity. Altogether, our data demonstrate the capacity of Dicer-independent miRNA biogenesis in *Drosophila* and provides mechanistic insight into the sorting and maturation of this atypical class of miRNA substrate.

## MATERIALS AND METHODS

### Plasmids

To generate *pUAST-DsRed-hsa-mir-144/451*, *hsa-mir-144/451* genomic fragment was PCR-amplified and cloned into the NotI/XhoI sites of UAS-DsRed plasmid. To generate *MT-DsRed-hsa-mir-144/451*, UAS-DsRed-*hsa-mir-144/451* was cut with EcoRI/XhoI to release the *DsRed-hsa-mir-144/451* fragment, which was cloned into the EcoRI/XhoI sites downstream of a copper-inducible metallothionein promoter in the pRmHa-3 plasmid.

To reprogram the mir-451 moiety of *mir-144/451* constructs, we designed appropriate oligonucleotides carrying mature sequences for various miRNAs and overlapping *mir-451* hairpin precursor. These primers were used in combination with either FP1 or TK polyA reverse primer in standard PCR reactions using *hsa-mir-144/451* construct as template. The two overlapping PCR products were purified and mixed together with EcoRI-digested *mir-144/451* plasmid, and a cold fusion reaction was carried out according to the manufacturer’s manual (System Biosciences).

For luciferase sensors, we designed oligonucleotide pairs containing two antisense miRNA sequences with 5′ NotI and 3′ XhoI-compatible ends. These target sequences were cloned into the NotI–XhoI sites downstream of the Renilla luciferase coding region in a modified psiCHECK2 vector (32), which carries an internal firefly luciferase gene for normalization (Promega). We used the same oligonucleotide pairs for cloning into the 3′ UTR of a *tubulin-GFP* P-element vector (33). The sensor sequences for the different miRNAs are listed in Supplementary Table S1 under ‘miRNA sensor primers’.

### *Drosophila* stocks

We generated multiple transgenic insertions of *UAS-DsRed-mir-144/451*, or versions in which *mir-451* was reprogrammed with the mature sequence of miR-19a-3p or miR-199a-3p, using standard Δ2-3 helper plasmid injections (BestGene, Inc.). For sensor assays, we also generated *tub-GFP-miR-144* and *tub-GFP-miR-451* sensor transgenes, and tested these in the background of spatially localized expression of *UAS-DsRed-mir-144/451* driven by *ptc-Gal4*, which is active at the anterior–posterior compartment boundaries of imaginal discs.

To drive ubiquitous expression of miRNA genes, balanced stocks of *UAS-DsRed-mir-144/451*, *UAS-DsRed-144/451:mir-19a* and *UAS-DsRed-mir-144/451:mir-199a* were crossed to the ubiquitously active *da-Gal4* driver. We extracted total RNA from DsRed-expressing third instar larvae of F1 progeny by TRIzol reagent and analyzed them by Northern blotting. To analyze the expression of *mir-144/451* in mutant backgrounds, we introduced *UAS-DsRed-mir-144/451* and *da-Gal4* into the following mutant backgrounds. We used the SM5-TM6B balancer to identify non-Tubby, mutant larvae of these genotypes.

**Table:** 

*Chr II control*	*w ; UAS-DsRed-144,451/+ ; da-Gal4/+*
*Chr III control*	*w ; +/+ ; UAS-DsRed-144,451/da-Gal4*
*pasha*	*w ; UAS-DsRed-144,451/+ ; da-Gal4, pasha[KO]/ pasha[KO]*
*drosha*	*w ; drosha[21K11]/Df(2 R)exel1055 ; UAS-DsRed- 144,451/da-Gal4*
*dicer-1*	*w ; UAS-DsRed-144,451/+ ; da-Gal4, dcr-1[Q1147X]/ Df(3 R)ED6096*
*dicer-2*	*w ; dcr-2[L811X] ; UAS-DsRed-144,451/da-Gal4*
*r2d2*	*w ; r2d2[1] ; UAS-DsRed-144,451/da-Gal4*
*ago2*	*w ; UAS-DsRed-144,451/+ ; da-Gal4, ago2[454]/ ago2[454]*

### *Drosophila* tissue culture

*Drosophila* cells were grown at 25°C in Schneider’s *Drosophila* Medium supplemented with 10% heat-inactivated FBS and 1% Penicillin-Streptomycin. S2R+ cells stably transformed with Flag-HA-AGO2 were previously described (21). To generate stable cells that express *mir-144/451*, S2R+ cells were co-transfected with MT-DsRed-*mir-144/451* and pCoHygro (10:1) with Effectene reagent (QIAGEN). Four days after transfection, cells were selected with 150 µg/ml hygromycin. Cells were collected every 3–4 days by centrifugation at 1200 rpm and resuspended with fresh medium containing 150 µg/ml hygromycin until cells started to grow. To induce expression of the miRNA construct, we added CuSO_4_ into the media to a final concentration of 2 mM for 2–3 days. The expression of DsRed and *mir-144/451* was examined by fluorescence microscopy and Northern blotting, respectively.

To investigate the effect of knockdowns in miRNA biogenesis, dsRNA soaking was performed in S2R+ cells. Approximately 500 bp fragments of target genes were amplified from *D. melanogaster w^−^* genomic DNA. The PCR-amplified fragments were cloned into the XhoI/XbaI sites of pLitmus (NEB), which contains opposing T7 promoters flanking the cloning site. T7 promoter-containing templates were generated by PCR amplification of pLitmus-dsRNA constructs with Litmus primer A+B, and dsRNA were synthesized using MEGAscript T7 Kit (Ambion). The primers used to amplify these regions are listed in Supplementary Table S1, ‘dsRNA primers’. To knockdown the expression of endogenous genes, 2.5 × 10^6^ S2R+/ cells were soaked with 15 µg dsRNA in six-well plate for 4 days and split into another six-well plate and soaked with 15 µg dsRNA for another 4 days. To induce *mir-144/451* expression in the knockdown experiments, 2 mM CuSO_4_ was added to the *MT-DsRed-mir-144/451* S2R+ stable cells on the fifth day of dsRNA soaking. For double knockdown experiments, 12 µg of each dsRNA was used; for triple knockdown experiments, 8 µg of each dsRNA was used. Knockdown efficiency of each dsRNA was analyzed by quantitative RT-PCR.

To assay the regulatory capacity of *mir-451*-type constructs in S2 cells, we co-transfected miRNA and sensor plasmids in 96-well plates for 72 h, and measured luciferase activities with Dual-Glo Luciferase Assay System (Promega).

### Western and Northern blotting

To prepare total cell lysates, cell pellets were resuspended in 5× cell pellet volume of IP/Extract buffer [150 mM NaCl, 2 mM MgOAc_2_, 20 mM Tris–HCl (pH 7.5), 5% Glycerol, 0.5% NP-40, 2 mM DTT, Complete-mini EDTA-free] and incubated on ice for 30 min. Cell lysates were cleared by centrifugation at 14 000 rpm for 10 min at 4°C. Protein samples were separated by SDS–PAGE and transferred to PVDF membrane with CAPS transfer buffer at 1.5 mA/cm^2^ for 45 min in the cold room. Membranes were blocked with 5% non-fat milk in TBST [150 mM NaCl, 30 mM Tris–HCl (pH 7.5), 2.5‰ Tween-20] for 1 h at room temperature (RT), incubated with primary antibodies with gentle agitation overnight at 4°C, washed three times for 5 min each with TBST, incubated with HRP-conjugated secondary antibodies for 1 h at RT, washed three times for 5 min each with TBST. The signals were detected with ECL Plus Western Blotting Detection Reagents (GE Healthcare Life Sciences).

Total RNA was extracted from cultured cells with Trizol (Life Technologies). RNA samples were separated on 20% urea polyacrylamide denaturing gels (National Diagnostics), transferred to GeneScreen Plus (Perkin Elmer), crosslinked using UV treatment and probed with γ-^32^P-labeled DNA or LNA oligonucleotides antisense to the small RNAs of interest at 45°C overnight. For DNA probes, the membranes were washed with 2× SSC/0.1% SDS at 45°C for four times, 15 min each time. For LNA probes, the membranes were washed with 2× SSC/0.1% SDS for three times and 0.2× SSC/0.1% SDS for two times at 45°C. The signals were exposed to Imaging Plate (Fujifilm). The sequences of the probes are listed in Supplementary Table S1 ‘Northern probes’.

To analyze Ago-associated RNA, we used an S2 cell line stably transformed with a FLAG-HA-AGO2 genomic construct (21,23). Cell lysates were cleared and then incubated with Dynabeads Protein G (Life Technologies) conjugated with AGO1 (ab5070, Abcam) or FLAG (M2, Sigma) antibodies (Supplementary Material). The Ago-associated RNA was extracted with 200 µl 0.4 M NaCl and 200 µl phenol/chloroform/isoamyl alcohol (25:24:1). The aqueous phase was ethanol precipitated at −20°C overnight. The RNA was analyzed as described above.

### miR-451 *in vitro* processing assays

We subjected synthetic 42-nt *pre-mir-451* RNA oligo (IDT) to 5′ phosphorylation using polynucleotide kinase and [γ-^32^P] ATP. We removed unincorporated [γ-^32^P] ATP with Illustra MicroSpin G-25 Columns (GE Healthcare). The radiolabeled RNA oligo was then separated on a 20% urea polyacrylamide denaturing gel, and RNA bands of the correct size were excised, crushed and extracted overnight in 400 mM NaCl at 4°C. The gel slice homogenates were collected by micropore filter (Ultrafree-MC, 0.22 μm, Millipore) and precipitated by 2.5 volumes of 100% ethanol and 20 μg glycogen at −20°C for 3 h. The RNA pellets were washed with 70% ethanol and resuspended with 500 μl oligo buffer [100 mM KOAc, 10 mM Tris–HCl (pH 8.0), 1 mM EDTA]. To fold the *pre-mir-451* hairpin, the RNA was denatured at 95°C for 5 min followed by immediate cooling on ice for >10 min. RNA was concentrated by Vivaspin 500 MWCO 3000 (GE Healthcare Life Sciences) until the volume was <50 μl. The amount of radioactivity in the labeled samples was measured by a liquid scintillation counter.

To perform the Ago protein cleavage assay, 2000 cpm γ-^32^P-labeled *pre-mir-451* RNA substrate was mixed with AGO1-immunoprecipitates from ∼1 mg S2 cells in 25 µl reaction buffer (20 mM HEPES-KOH pH 7.3, 100 mM KOAc, 2 mM MgOAc_2_, 2 mM DTT, 30 U RNaseOUT). The Ago cleavage reaction was carried out at RT for ∼2 h, followed by wash for two times with the reaction buffer. To test the *ac-pre-mir-451* 3′ trimming activity in different lysates, ∼100 µg total lysates from S2 cells or fly ovary tissues were incubated with the washed beads at RT for 2 h in the presence of 30 U RNaseOUT (Roche). After two times of wash with reaction buffer, RNA was extracted and analyzed as previously described.

### Small RNA library cloning and analysis

dsRNA (75 µg total) knockdown experiments were carried out in 10 ml *MT-DsRed-mir-144/451* S2R+ cells for 8 days, with CuSO_4_ added on the fifth day to induce the miRNA construct. The cells were lysed with IP/Extract buffer, and cleared by centrifugation at 14 000 rpm for 15 min at 4°C. The extracted RNA was resuspended in water and spiked in with 10 index small RNA oligos (see Supplementary Table S2) along with ^32^P-labeled 19 and 29 nt oligos bearing PmeI sites. AGO1-associated RNA was extracted largely as previously described (25), using randomized 3′ adaptor (5′ rAppNNNNTGGAATTCTCGGGTGCCAAGG/ 3ddC/) and randomized 5′ adaptor (5′ GUUCAGAGUUCUACAGUCCGACGAUCNNNN). The ligated RNA products were reverse transcribed (RT primer 5′ GCCTTGGCACCCGAGAATTCCA) at 50°C for 1 h with SuperScript III (Life Technologies) and heated to 70°C for 15 min. The cDNAs were amplified by standard Taq polymerase (NEB) using RP1 forward primer (5′ AATGATACGGCGACCACCGAGATCTACACGTTCAGAGTTCTACAGTCCGA) and different 5′ index primers RPI (x) (NEBNext® Multiplex Oligos, Illumina) for each sample, followed by digestion of the index oligos by PmeI at 37°C for 3 h.

Small RNA sequencing reads were trimmed and mapped to the dm3 genome as described (34). Reads mapping to different genomic categories were identified with the bedtools intersect program (35), using annotations from Flybase v5.40 and mirBase v17, including miR-451. To identify candidate mir-451 like loci, the genome was scanned for regions with AGO1-IP reads, using the following criteria: Regions with at least 40 read coverage over 20 nt, where at least 75% of the reads map to the same strand and with free folding energy of less than or equal to −10 kcal/mol (as predicted by RNAfold).

## RESULTS

### *mir-451-*class substrates generate functional regulatory RNAs in *Drosophila*

**Figure 1.**
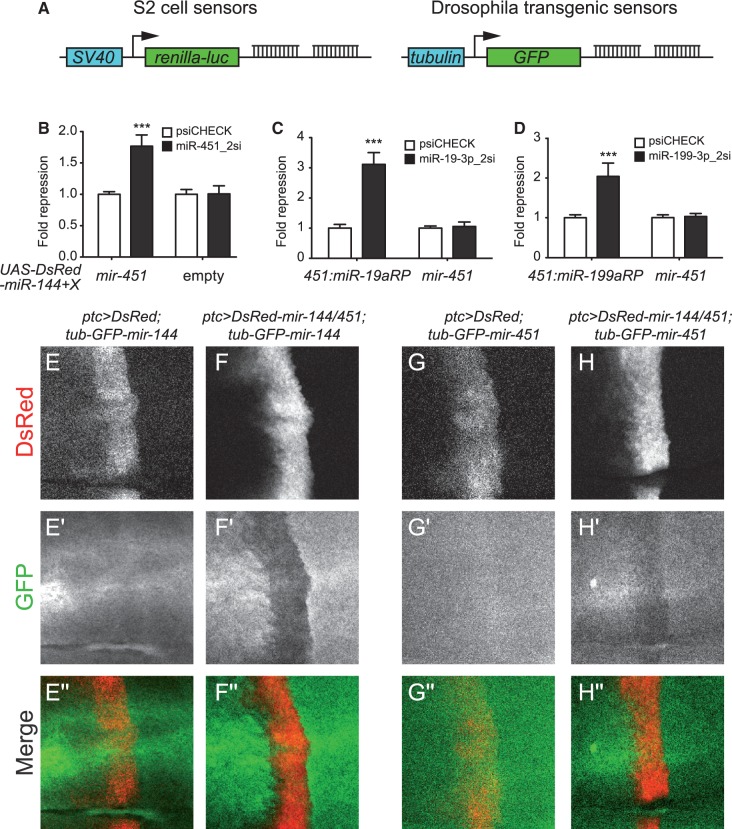
*mir-451*-type substrates generate functional regulatory RNAs in *Drosophila*. **(A)** Sensor design. We assayed renilla luciferase sensors driven by the SV40 early promoter in S2 cells, and GFP sensors driven by the tubulin promoter in transgenic *Drosophila*. All sensors contained two sites fully complementary to the mature miRNA under assay. **(B–D)** Sensor assays in S2 cells that were transfected with *ub-Gal4*, *UAS-DsRed-mir-144/451* or variants in which the *mir-451* moiety was reprogrammed (RP) with miR-19a-3p or miR-199a-3p, and psiCHECK sensors bearing control firefly luciferase and a test renilla luciferase bearing two complementary sites for the indicated miRNA. We observed specific target repression by miR-451 (B), 451:miR-19aRP (C) and 451:miR-199aRP (D) constructs. Sensor tests using empty or non-cognate miR-451 constructs provided further evidence for the specificity of repression that we observed. **(E–H)** Sensor assays in third instar wing imaginal discs. Shown are the central wing pouch regions of discs that express *ptc-Gal4*, *UAS-DsRed* or *UAS-DsRed-mir-144/451* (in red), and either *tub-GFP-mir-144* or *tub-GFP-mir-451* (in green). The loss of GFP protein in DsRed+ domains, relative to adjacent DsRed- tissue, indicates functional repression. Thus, DsRed was inert on the *GFP-miR-144* sensor (E), but this was repressed by expression of *mir-144/451* (F). Likewise, DsRed was inert on the *GFP-miR-451* sensor (G), but this was repressed by expression of *mir-144/451* (H).

Since *mir-451*-class substrates have not been identified outside of vertebrates, we were interested to determine if *Drosophila* was capable of utilizing this non-canonical type of substrate. We considered the most salient initial tests to be functional repression assays, which we performed using luciferase sensors in S2 cells and GFP sensors in transgenic animals (Figure 1A). We cloned a human *mir-144/451* pri-miRNA fragment into a *UAS-DsRed* vector for expression in *Drosophila*; the *mir-144* moiety serves a canonical miRNA control for these experiments. We transfected this with *ub-Gal4* activator plasmid into S2 cells, and assayed for repression of a renilla luciferase ‘perfect’ miR-451 sensor that we had validated in human cells (8). This construct induced specific miR-451-dependent repression of its sensor (Figure 1B), suggesting that Ago-mediated miRNA biogenesis is operational in *Drosophila* cells. We note that this assay does not distinguish the relative contributions of the two *Drosophila* Argonaute proteins, both of which have Slicer activity, but earlier quantitative analyses indicate that they may mostly report on the activity of AGO2 (22).

Although the only endogenous substrate of this pathway currently known is miR-451 itself, we showed that its backbone constitutes a flexible platform for the production of diverse Dicer-independent miRNAs from plasmid constructs (8,11). We therefore tested constructs in which we reprogrammed miR-451 with other mature miRNA sequences, such as miR-19a-3p and miR-199a-3p. Indeed, we successfully detected the ability of both constructs to repress cognate sensors (Figure 1C and D). Thus, the capacity for regulation by *mir-451*-type substrates in *Drosophila* is flexible to hairpin sequence.

We performed more stringent regulatory tests of *mir-451* in the animal. We generated *UAS-DsRed-mir-144/451* transgenes, and tested their ability to repress ubiquitously expressed *tub-GFP* sensor transgenes linked to either miR-144 or miR-451 complementary sites. We activated the miRNAs using *ptc-Gal4*, which is active in a stripe of cells at the anterior–posterior compartment boundary of the wing imaginal disc. By focusing on the wing pouch region, which is relatively flat, we can obtain a clear read-out of GFP sensor expression in miRNA/DsRed-positive cells, compared to flanking DsRed-negative control cells. We first assessed canonical miR-144. Control expression of *UAS-DsRed* did not affect the GFP-miR-144 sensor, but similar expression of *UAS-DsRed-mir-144/451* induced strong repression (Figure 1E and F). Therefore, this human miRNA construct is active in *Drosophila*. Similar assays of GFP-miR-451 sensor showed that it was specifically down-regulated by expression of *UAS-DsRed-mir-144/451* (Figure 1G and H). Although miR-451 was not as potent as miR-144, the demonstration of regulatory activity of miR-451-type substrates in *Drosophila* cells and animals encouraged us to examine their mechanism of biogenesis.

### Biogenesis of bulk *mir-451-*class hairpins is arrested at the Ago cleavage stage

**Figure 2.**
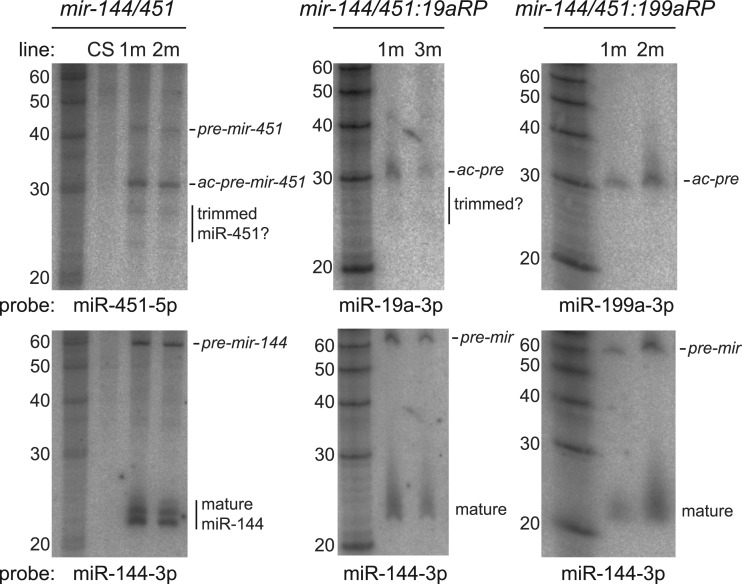
Bulk maturation of *mir-451*-type substrates in *Drosophila* arrests as ∼30 nt species. We performed Northern blotting of third instar larvae bearing *da-Gal4* and *UAS-DsRed-mir-144/451*, or variants in which the *mir-451* moiety was reprogrammed (RP) with miR-19a-3p or miR-199a-3p. We assayed two independent transgenic insertions for each construct, and used Canton S (CS) as a control genotype. Although a modest amount of potentially trimmed <30 nt species was seen, the majority of signals for miR-451 or reprogrammed miR-451 constructs were at ∼30 nt. Control blotting for *mir-144* showed its expected accumulation in all transgenic animals.

We collected RNA from larvae in which *UAS-DsRed-mir-144/451* was activated ubiquitously using *da-Gal4*, and assessed the accumulation of small RNAs from this transgene using Northern blotting. *Drosophila* lacks orthologs of miR-144 or miR-451, allowing us to assign hybridizing bands to the introduced constructs. As shown in Figure 2, these constructs generated both ∼58 nt *pre-mir-144* hairpin and mature ∼21–23 nt miR-144 species. We also observed accumulation of ∼42 nt *pre-mir-451* and ∼30 nt *ac-pre-mir-451*, with the latter present at ∼2.8-fold greater levels than the former. However, mature <30 nt miR-451 species were barely detected in total RNA samples.

We performed similar Northern blotting for RNAs generated from *mir-144/451* transgenes in which miR-451 was reprogrammed with miR-19a-3p or miR-199a-3p. In these tests, we similarly observed predominant accumulation of ∼30 nt ac-pre-miRNAs, with little or no fully matured species (Figure 2). We tested two independent insertions of each miR-451-class transgene, and observed similar results in all cases. Overall, these data indicate that 3′ resection of Ago-cleaved miR-451-type substrates is not as effective in *Drosophila* compared with vertebrates.

### Biogenesis of *mir-451* requires the Microprocessor complex but is stimulated by loss of the Dcr-1/Loqs complex

We analyzed the dependence of miR-451 on the canonical miRNA pathway machinery using dsRNA-mediated knockdowns in S2 cells (36). In initial tests, we found that the transfection efficiencies of cells that had been depleted of different small RNA factors was variable (data not shown). Therefore, we generated a stable S2R+ line bearing a copper-inducible *mir-144/451* expression construct (*MT-DsRed-mir-144/451*), which enabled more uniform measurements across a panel of conditions. We soaked these cells in dsRNA for 4 days, and then replaced the media with fresh dsRNA for another 4 days to ensure strong knockdown. We induced *mir-144/451* expression on the fifth day and collected RNA for analysis on the eighth day of dsRNA treatment.

**Figure 3.**
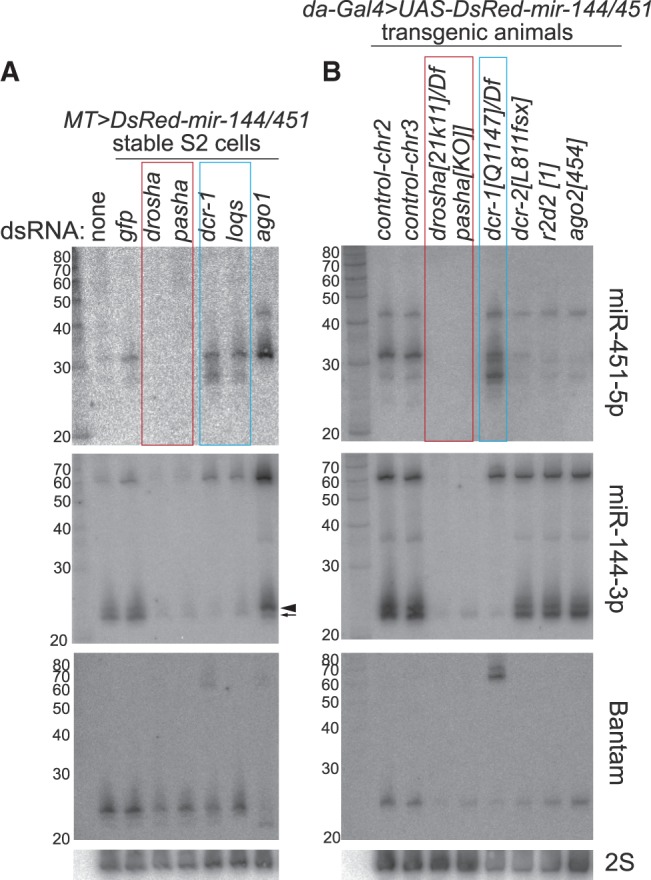
Genetic requirements for miRNA and siRNA factors to mature miR-451. **(A)** Knockdown assays in S2 cells stably transformed with *MT-DsRed-mir-144/451*. Following dsRNA-mediated depletion of the indicated factor, expression of *mir-144/451* was induced using copper, and the resulting RNAs were analyzed by successive rounds of Northern blotting and stripping. Biogenesis of all miR-451 small RNAs was abolished by knockdown of the nuclear miRNA factors Drosha and Pasha, but knockdown of Dicer-1 and Loqs enhanced the accumulation of <30 nt miR-451 species. Depletion of AGO1 enhanced the accumulation of ∼42 and ∼30 nt miR-451 species. The canonical loci miR-144 and bantam exhibit the expected dependencies on these miRNA factors. Note though that, depletion of AGO1 results in loss of most bantam signals, whereas a larger isoform of miR-144 accumulates (compare arrowhead versus arrow); subsequent analysis indicated that the larger miR-144 isoform is loaded into AGO2 complex. **(B)** Expression of *da-Gal4*, *UAS-DsRed-mir-144/451* in third instar larvae of the indicated genotypes. Control lines are insertions of *UAS-DsRed-mir-144/451* on different chromosomes. The insertion used for each genotype is on the autosome that does not carry the mutant allele (chr2 insertion for *pasha*, *dcr-1* and *ago2*, chr3 insertion for *drosha*, *dcr-2* and *r2d2*). As in S2 cells, mutations of *drosha* and *pasha* eliminate the accumulation of processed miR-451 species, whereas mutation of *dcr-1* enhances its maturation. Mutants of the RNAi factors *dcr-2*, *r2d2* and *ago2* selectively reduced the ∼30 nt miR-451 species.

As in vertebrates (8–10), the accumulation of all processed forms of miR-451 was dependent on Drosha and its dsRBD partner DGCR8/Pasha (Figure 3A). Knockdown of these factors eliminated hybridization to miR-451 probes, and also strongly depleted mature and hairpin forms of miR-144. Interestingly, not only was the biogenesis of miR-451 independent of the pre-miRNA-specific RNase III enzyme Dcr-1, depletion of this factor actually enhanced the accumulation of <30 nt miR-451 species (Figure 3A). Consistent with this, depletion of the Dcr-1 dsRBD cofactor, Loquacious (Loqs), also stimulated the accumulation of <30 nt miR-451 species (Figure 3A).

We validated these findings using null mutants of core miRNA biogenesis factors. To do so, we introduced *da-Gal4* and *UAS-DsRed-mir-144/451* transgenes into *drosha[21K11]*, *pasha[KO]* and *dcr-1[Q1147]* mutants and/or deficiencies, and isolated RNA from homozygous or hemizygous mutant larvae. These genotypes confirmed the complete dependence of both miR-451 and miR-144 maturation on nuclear miRNA factors. On the other hand, while mutation of *dcr-1* blocked the maturation of miR-144, it concomitantly enhanced the maturation of miR-451 (Figure 3B).

We conclude from these experiments that full 3′ resection of miR-451 is possible in *Drosophila*, but that this process is in competition with endogenous canonical pre-miRNA hairpins. Reduction of canonical hairpins generated by Dicer-1 and Loqs can thus enhance the availability of *pre-mir-451* intermediates for maturation.

### Maturation of mir-451 is also influenced by the RNAi machinery

We followed up this analysis by examining the requirement of RNAi factors for miR-451 biogenesis. As depletion of RNAi factors in S2 cells resulted in cell toxicity, possibly due to unleashing of persistent viruses (37), we moved directly to analyze the appropriate null mutant flies. We introduced *da-Gal4* and *UAS-DsRed-mir-144/451* transgenes into the homozygous viable backgrounds of *dcr-2[L811fsx]* and *r2d2[1]*, and isolated larvae for Northern blotting. As expected, these backgrounds did not affect the maturation of miR-144. However, we observed specific depletion of ∼30 nt miR-451 species, whereas ∼42 and <30 nt species were relatively little affected (Figure 3B).

Taken together with the miRNA pathway manipulations, these data indicated complex genetic requirements and impact of both canonical miRNA and siRNA factors on the biogenesis of *mir-451* in *Drosophila*. In particular, there are several sizes of miR-451 species (42, 30 and <30 nt), whose accumulations are differentially affected by miRNA and siRNA factors.

### Interplay of miRNA and siRNA sorting pathways for *mir-451* biogenesis

We hypothesized that the complex genetic requirements for miR-451 maturation might reflect the possibility that short hairpins assort into both miRNA and siRNA pathways, and thus access both Argonaute effectors. In mammalian cells, multiple Ago proteins can load *pre-mir-451* hairpins, but only those hairpins that load the sole vertebrate Slicer Ago2 are capable of maturing miR-451 further. Although the siRNA effector AGO2 is the dominant Slicer Argonaute in *Drosophila* (22), the miRNA effector AGO1 also exhibits detectable cleavage activity that can be programmed by endogenous miRNAs and siRNAs (13,25).

We examined the maturation of *MT-DsRedmir-144/451* following knockdown of *AGO1* in S2 cells. Interestingly, this condition nearly abolished the accumulation of <30 nt miR-451 species, and increased the amount of 30 nt products (Figure 3A). We were unable to examine *ago1* mutants, which are embryonic lethal (38) and prohibitive to obtain sufficient mutant material for Northern blotting. However, we could test this in *ago2* mutants, which are viable. We recombined *da-Gal4* and *UAS-DsRed-mir-144/451* transgenes into *ago2[454]*, and observed that this mutant behaved similarly to *dcr-2* and *r2d2*, in that 30 nt miR-451 species were specifically depleted (Figure 3B). Therefore, the loss of AGO1 may permit the re-sorting of a subpopulation of *pre-mir-451* hairpins to AGO2, but the reciprocal loss of AGO2 does not effectively permit re-sorting to AGO1, possibly because they are out-competed by miRNA hairpins.

**Figure 4.**
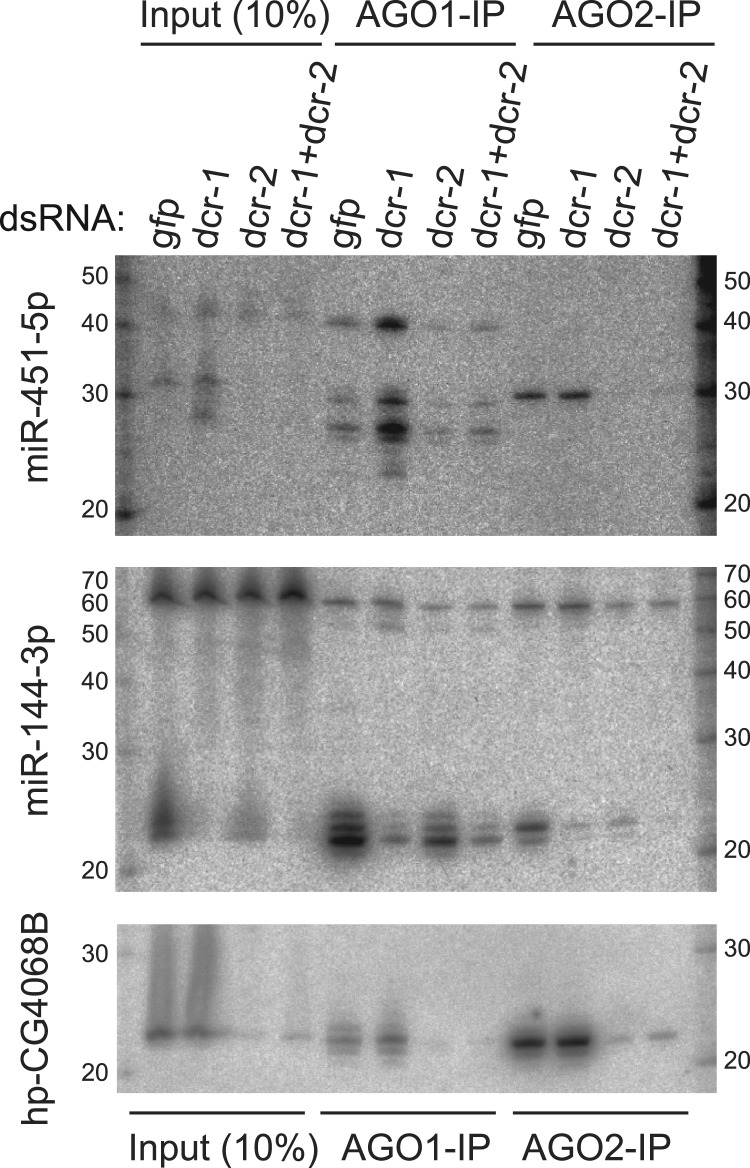
Distinct requirements of two Dicer proteins for miR-451 sorting to the two Ago proteins. We depleted stable Flag-AGO2 S2R+ cells of the indicated factors using dsRNA, and then transfected them with *ub-Gal4* and *UAS-DsRed-mir-144/451*. We then immunoprecipitated AGO1 and Flag-AGO2 complexes and examined their associated small RNAs using Northern blotting. Consistent with previous results, the input lanes show predominant accumulation of ∼42 nt (*pre-mir-451*) and ∼30 nt (*ac-pre-mir-451*) species in control knockdown cells, while the accumulation of <30 nt trimmed miR-451 species was enhanced by depletion of Dcr-1 and the accumulation of ac-pre-mir-451 species was eliminated by depletion of Dcr-2. Analysis of Ago complexes revealed that AGO1 associates with ∼42, ∼30 and <30 nt miR-451 species, whereas AGO2 selectively accumulates ∼30 nt miR-451 species. Note that the *ac-pre-miR-451* species in AGO2 are slightly larger than the comparable species in AGO1; we later show that *ac-pre-miR-451* species in AGO2 are 3′ modified by Hen1 methyltransferase (Figure 5). The Dcr-1-dependent canonical miRNA miR-144 preferentially accumulates in AGO1 complex while the Dcr-2-dependent endo-siRNA hp-CG4068B preferentially accumulates in AGO2 complex, validating the knockdown and AGO-IP procedures. Note though that the largest miR-144 isoform preferentially associates with AGO2, and its accumulation therein is dependent on Dcr-2.

To examine this more directly, we sequentially purified AGO1 and Flag-AGO2 complexes from S2R+ cells stably expressing FLAG-HA-AGO2, and examined their immunoprecipitates for different small RNAs. Mature miR-144 was preferentially associated with AGO1 relative to AGO2, as expected for a canonical miRNA (Figure 4). We note that the dominant miR-144 species in AGO2 was 1 nt longer than in AGO1, in line with our finding that depletion of *ago1* causes a slightly larger mature miR-144 species to accumulate (Figure 3A). By comparison, the endo-siRNA product hp-CG4068B preferentially associated with AGO2, validating the purity of our effector complexes (Figure 4).

Consistent with earlier results, total RNA from cells treated with *gfp* dsRNA predominantly showed the ∼42 and ∼30 nt bands (Figure 2). Interestingly, AGO1-IP complexes from these cells contained the *pre-mir-451* and a species that appeared to be slightly shorter than the 30 nt species seen in total RNA; moreover they also contained shorter miR-451 species corresponding to 3′ resected mature miR-451 (Figure 4). Since S2 total RNA samples only modestly accumulated <30 nt miR-451 species, we infer that these are rarer products that were enriched by purification of AGO1 complexes. Likewise, the mild excess of <30 nt species over ∼42 nt species in AGO1 complexes suggested that the majority of 42 nt species observed in total RNA are not associated with AGO1. In contrast, AGO2 complexes from these cells contained exclusively the ∼30 nt species, which migrated slightly slower than the largest <30 nt species in AGO1 complex.

These data allow us to infer that the various mir-451-hybridized products actually correspond to a mixture of distinct populations: (i) a ∼42 nt Drosha/Pasha-cleaved hairpin that is in transit for further maturation, (ii) a minor population of ∼42 nt species that associates with AGO1 and can be cleaved into ∼30 nt hairpins and 3′ resected into <30 nt species and (iii) a major population of 30 nt species that are associated with AGO2. Moreover, these data indicate that full maturation of miR-451 is possible provided that its pre-miRNA is directed into AGO1; hairpin loading to AGO2 results in efficient cleavage, but this product is apparently blocked for further maturation.

### Influence of the two *Drosophila* Dicers on *mir-451* sorting and maturation

We next tested the impact of depleting Dicer-class enzymes on these sorting patterns. Knockdown of *dcr-1* strongly enhanced the steady-state association of all three forms of *mir-451* species with AGO1 (Figure 4). Since even the 42 nt species increased in AGO1 complexes, we infer that *pre-mir-451* may load AGO1 more efficiently when it is no longer competing with loading of endogenous miRNA duplexes. We observed a minor decrease in all *mir-451* species associated to AGO1 upon depletion of *dcr-2*, perhaps suggesting that it plays a role in trafficking of *pre-mir-451* hairpins. Reciprocally, the pure 30 nt miR-451 products in AGO2 complexes were unaffected by depletion of *dcr-1*, while single *dcr-2*-KD or double *dcr-1/2*-KD nearly abolished its accumulation in AGO2 (Figure 4). Since 42 nt *pre-mir-451* still associates with AGO1 in *dcr-2*-KD conditions, we infer that miR-451 processing is independent of Dcr-2, but that it requires Dcr-2 in its capacity as an AGO2 loading factor (18).

In contrast to miR-451, mature miR-144 species in AGO2 complexes were strongly depleted in all three conditions (*dcr-1*-KD, *dcr-2*-KD and *dcr-1/2*-KD), since it should require Dcr-1 for pre-miRNA cleavage and Dcr-2 for loading to AGO2 (Figure 4). The endo-siRNA hpCG4068B associated with AGO2 to similar levels in cells treated with *gfp* and *dcr-1* dsRNAs, as expected for an endo-siRNA. Altogether, these studies demonstrate distinct requirements for Dicer enzymes for accumulation of small RNAs from a endo-siRNA hairpin, a canonical miRNA hairpin and a Dicer-independent hairpin in the RNAi effector AGO2.

### Depletion of Hen1 methyltransferase permits 3′ resection in AGO2

**Figure 5.**
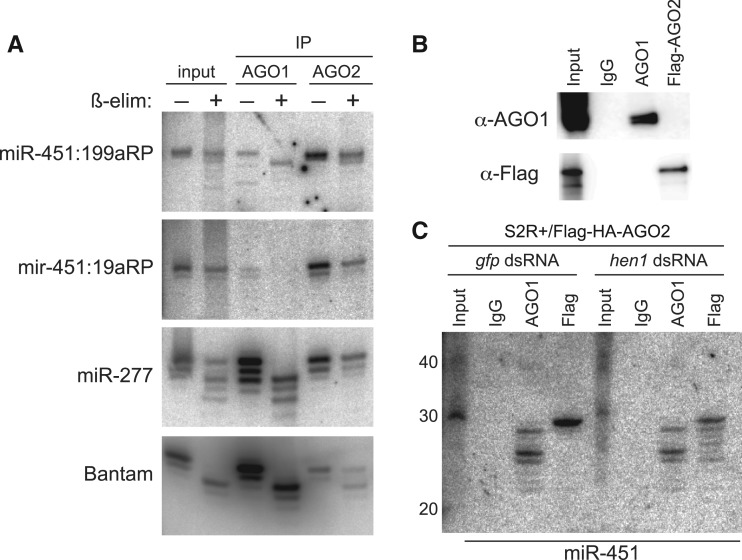
Hen1-dependent modification of *ac-pre-mir-451* species in AGO2 complexes. **(A)** We analyzed total RNAs before and after ß-elimination. Small RNAs that have reacted exhibit increased mobility and/or decreased apparent level. Most of the pool of the canonical miRNA bantam, which is preferentially loaded in AGO1, is amenable to ß-elimination. A substantial portion of miR-277, which is sorted into both AGO1 and AGO2, was not ß-eliminated. The bulk of small RNAs from *mir-144/451* constructs reprogrammed with miR-199a-3p or miR-19a-3p, which accumulate as ac-pre-miRNAs, were resistant to ß-elimination. **(B)** Western blotting confirms the selective isolation of AGO1 and AGO2 complexes. **(C)** We soaked S2R+ cells stably expressing Flag-HA-AGO2 with *gfp* or *hen1* dsRNAs, then transfected them with *ub-Gal4* and *UAS-DsRed-mir-144/451*. As before, AGO1 complexes normally contain <30 nt miR-451 species while AGO2 complexes strictly contain 30 nt *ac-pre-mir-451*. However, depletion of *hen1* permits the accumulation of 3′ trimmed miR-451 species in AGO2.

Single-stranded RNAs in AGO2, but not in AGO1, are modified at their 3′ ends by the Hen1 methyltransferase (39). This modification renders matured RNAs in AGO2 resistant to ß-elimination, while mature RNAs in AGO1 are sensitive resulting in their increased mobility. For example, the bulk population of the canonical miRNA bantam is sensitive to ß-elimination, and this is due to its dominant sorting to AGO1. On the other hand, miR-277 is an atypical miRNA that is sorted into both AGO1 and AGO2 effectors (22), for which the AGO1-loaded population is sensitive to ß-elimination while the AGO2-loaded population is resistant (Figure 5A).

Consistent with our observations of preferential loading to AGO2, bulk ∼30 nt *ac-pre-mir-451* species in total RNA were resistant to ß-elimination (Supplementary Figure S1). In addition, analysis of *mir-451* species in AGO2-IP and AGO1-IP complexes showed that the former were sensitive while the latter were resistant (Supplementary Figure S1). We performed similar analysis using *mir-451* constructs reprogrammed with mature miR-199a or miR-19a, and found that bulk ∼30 nt small RNAs from both constructs were resistant to ß-elimination, and that this was due to their dominant sorting to AGO2 (Figure 5A). Western analysis confirmed the selectivity of the AGO1 and Flag-AGO2 immunoprecipitations (Figure 5B).

We inferred that the free hydroxyl groups on the 3′ end of *ac-pre-mir-451* in AGO1 complexes permitted its resection. We therefore asked whether depletion of *hen1* could promote miR-451 resection in AGO2 complexes. This was indeed the case: cells treated with control *gfp* dsRNA maintained a single 30 nt *ac-pre-mir-451* in AGO2-IP, but treatment with *hen1* dsRNA permitted a ladder of shortened miR-451 species to accumulate in AGO2 complexes (Figure 5C).

These data indicate that 3′ resection obligately occurs within a mature Ago complex, and that miR-451 intermediates and products are not exchanged between Ago complexes. This notion is supported by the observation of distinctly-sized miR-451 species in AGO1 and AGO2 (Figure 4). Moreover, they indicate that the modest processing of *mir-451* observed in wildtype cells is a consequence of the separation of siRNA and miRNA pathways in *Drosophila*, such that default sorting of *pre-mir-451* into AGO2 leads to its 3′ modification and blockage from further resection. Enhancement of *pre-mir-451* loading into AGO1 by genetic means (e.g. by loss of Dcr-1 or Loqs), or prevention of 3′ modification of *ac-pre-mir-451* in AGO2 (e.g. by loss of Hen1), are both compatible with full maturation of miR-451.

### Depletion of Nibbler 3′–5′ exoribonuclease enhances 3′ resection of miR-451

The inhibition of *ac-pre-mir-451* maturation by Hen1-dependent 3′ modification suggested that its trimming is performed by a 3′–5′ exoribonuclease, as opposed to an endoribonuclease. We attempted to identify a cognate factor by depleting candidate 3′–5′ exoribonucleases in S2 cells, and assaying for effects on miR-451 maturation. We soaked *MT-DsRed-mir-144/451* cells with dsRNAs against various RNases, then induced miRNA expression and analyzed the resultant RNAs by Northern blotting.

**Figure 6.**
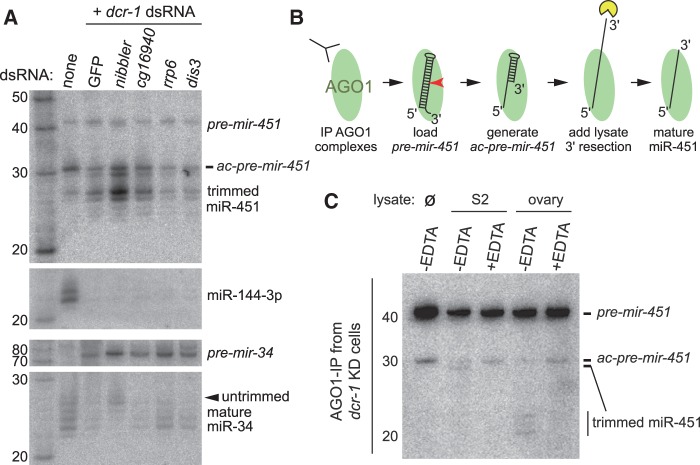
3′ resection of miR-451 is antagonized by Nibbler and promoted by a soluble EDTA-sensitive ribonuclease. **(A)** We treated S2R+ cells stably transformed with *MT-DsRed-mir-144/451* with dsRNAs against the indicated RNases, and processing of miR-451 was enhanced by co-depletion of *dicer-1*. Following treatment with copper to induce miRNA expression, we analyzed total RNAs using Northern blotting. Interestingly, depletion of the 3′–5′ exoribonuclease Nibbler, which shortens the 3′ tails of ‘long’ miRNAs in AGO1 complexes, actually enhanced the 3′ resection of miR-451. Effective depletion of Dicer-1 was evidenced by the loss of mature miR-144, accumulation of *pre-mir-34*, and shift in the size distribution of the remaining mature miR-34 to its longest isoform. **(B)** Scheme for *in vitro* processing assay for the miR-451 3′ resectase. **(C)** Incubation of 5′ radiolabeled *pre-mir-451* with AGO1 complexes immunopurified from dsDcr-1 cells generates a detectable amount of stable 30 nt *ac-pre-mir-451* species. Following treatment with S2 cell lysate, we observed a modest amount of 3′ trimming (∼1 nt); this was blocked by pretreatment of lysate with EDTA. Treatment with ovary lysate yielded a greater amount of trimmed products, and this activity was again EDTA sensitive.

These tests did not identify any individual ribonuclease whose depletion caused a substantial effect on miR-451 3′ trimming (data not shown). To sensitize these tests, we then performed double knockdowns of exoribonucleases with *dcr-1*, whose depletion enhances the signals for mature miR-451 in total RNA (Figures 3 and 4). However, these tests did not clearly yield a condition under which *ac-pre-mir-451* preferentially accumulated (Supplementary Figure S2), as might be expected upon loss of the 3′ resectase. For example, although the RNA exosome removes the unstructured ends of a subclass of splicing-derived hairpins that require 3′ trimming to generate the pre-miRNA substrate, since precursor 3′ tailed mirtrons accumulate following loss of the exosome (40). However, the exosome RNases Rrp6 and Dis3 did not appear to be required for miR-451 maturation, and neither was the Dis3-related protein CG16940 (Figure 6A). It is unclear whether the nucleases tested were insufficiently depleted, whether the relevant nuclease was not tested, or whether there might be redundant activities. However, these tests did yield an unexpected finding, namely that depletion of Nibbler (30,31) substantially enhanced the maturation of miR-451. Whereas little <30 nt miR-451 species was in total RNA from control *gfp/dcr-1* dsRNA-treated S2 cells, these were more abundant in cells codepleted of *dcr-1* and *nibbler* (Figure 6A).

The Nibbler 3′–5′ exoribonuclease mediates broad 3′ trimming of mature miRNAs in AGO1 complexes (30,31), especially in cases where the species produced by Dicer-1 cleavage are relatively long (41). For example, miR-34 is initially produced as a 24 nt product, but Nibbler resects 3–4 nt from its 3′ end. We verified that in our *nibbler* knockdowns, miR-34 was stabilized at the length generated by Dicer-1 cleavage (Figure 6A, miR-34 blot, arrowhead). We performed further single and double knockdowns of *gfp*, *dcr-1* and *nibbler* dsRNAs to demonstrate that *dcr-1/nibbler* double depletion synergize in promoting miR-451 maturation (Supplementary Figure S3). Moreover, knockdown of *nibbler* alone could enhance the accumulation of 3′-resected miR-451 (Supplementary Figure S3). Since Nibbler does not correspond to the miR-451 ‘Resector’, these findings imply the existence of multiple, potentially competing, ribonucleases that can shape the 3′ ends of different miRNA substrates in *Drosophila*.

### *In vitro* recapitulation of the 3′ resection of AGO1-cleaved *pre-mir-451*

Since we were not able to identify the miR-451 3′ trimming enzyme genetically, we attempted to gain insight into its properties using a biochemical approach. Inspired by the recent *in vitro* recapitulation of piRNA 3′ end formation (42), we set up an analogous assay for miR-451 processing (Figure 6B). We first immunoprecipitated AGO1 from S2 cells, and loaded it with 5′ radiolabeled synthetic *pre-mir-451*. However, we were not able to observe *in vitro* cleavage to generate *ac-pre-mir-451* (Supplementary Figure S4A). Subsequent reactions showed that some *pre-mir-451* associated with control Myc-Ab beads despite extensive washing (Supplementary Figure S4B). Therefore, a fraction of visualized substrates were not genuinely loaded in AGO1, which might obscure our ability to detect *mir-451* processing.

Based on our observation that loss of canonical miRNA biogenesis enhances *mir-451* maturation, we then isolated AGO1 complexes from S2 cells depleted of Dcr-1. Following loading of *pre-mir-451*, we were now able to observe the generation of 30 nt *ac-pre-mir-451* (Figure 6C, ‘no lysate’ lane). Even though the loss of Dcr-1 substantially decreases the steady-state level of AGO1 (43), these data support the interpretation that absence of canonical miRNA biogenesis enhances the assembly of AGO1:*pre-mir-451* complexes.

We then treated this with lysates of S2 cells or *Drosophila* ovaries. We observed only mild 3′ trimming in the S2 lysate, perhaps of a single nucleotide (Figure 6C). However, we obtained reasonably robust trimming using soluble ovary lysate, such that most of the 30 nt species was reduced to mature miRNA-sized species (Figure 6C). This activity was notable given that the piRNA ‘Trimmer’, which also exists in ovarian (silkworm) cells, is completely insoluble (42); thus, our assay may potentially detect the activity of a distinct resectase. We tested the dependence of this activity on divalent cations by supplementing the lysates with EDTA. Even though S2 lysate was only capable of removing 1 nt, this was blocked by EDTA. The more substantial 3′ trimming obtained with ovary lysate was also fully blocked by EDTA. Therefore, a soluble miR-451 3′ trimming activity can be detected *in vitro*, and it appears to act by a metal ion-dependent mechanism.

### Deep sequencing of AGO1 complexes from cells depleted of *dcr-1*

No endogenous *mir-451*-type substrates are yet known in *Drosophila*. We attempted to identify such loci using extant AGO1/2-IP small RNA data from S2 cells (27), ovaries (23,25) and heads (20), but did not find convincing evidence for *mir-451*-type loci (data not shown). Still, in light of the fact that conserved vertebrate miR-451 exhibits narrow tissue specificity, being largely restricted to red blood cells (44,45), it is conceivable that the few extant sources of data from *Drosophila* Argonaute complexes do not include the expression domain of a putative member of this class. Our studies provided a potentially useful lead, in that loss of Dicer-1 enhances the maturation of miR-451 in AGO1 complexes (Figure 4).

**Figure 7.**
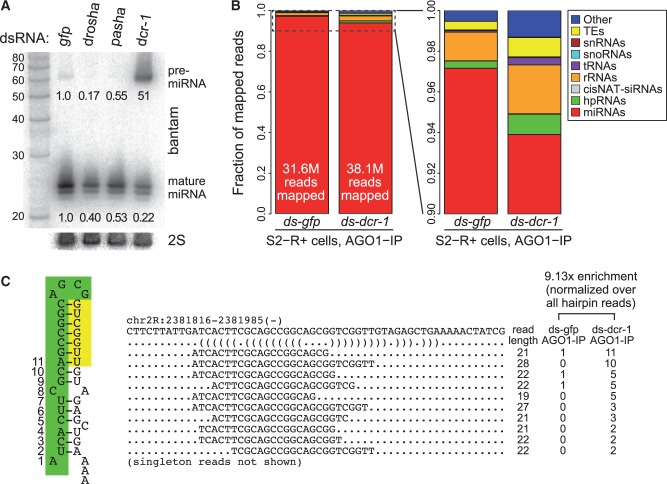
Analysis of AGO1-IP libraries from *gfp* and *dcr-1* knockdown conditions. **(A)** Validation that knockdown of miRNA biogenesis factors in *MT-DsRed-mir-144/451* S2 cells leads to strong defects in endogenous bantam maturation. **(B)** AGO1-IP libraries were constructed from the *gfp* and *dcr-1* knockdown conditions. Cursory analysis shows that the both datasets were largely composed of mature miRNAs, despite the direct evidence shown for strongly defective pre-miRNA dicing and loss of mature miRNA in these cells. However, closer examination reveals a substantial increase in non-miRNA reads in the *dcr-1* knockdown AGO1-IP library. **(C)** Example of a candidate *mir-451*-like short hairpin, associated with reads bearing a dominant 5′ end, mature reads that cross the terminal loop, other longer reads that extend into the 3′ arm, and strongly enhanced accumulation in the *dcr-1* knockdown AGO1-IP library.

We tested this notion by soaking *MT-DsRed-mir-144/451* S2R+ cells for two rounds of 4 days each with dsRNAs, and induced the miRNA construct on the fifth day of the regimen. Under these conditions, we confirmed substantial depletion of mature bantam and strong accumulation of pre-bantam (Figure 7A). We proceeded to generate AGO1-IP libraries from the *gfp* and *dcr-1* samples. Given that miRNAs were so strongly depleted, it was perhaps surprising that both libraries were mostly composed of mature canonical miRNAs (Figure 7B, left graphs). However, we can rationalize this result in light of our recent appreciation that loss of canonical miRNA biogenesis factors concomitantly destabilizes AGO1 protein (43). Thus, the process of purifying AGO1 complexes for library construction obscures the fact that the absolute amount of miRNAs (and AGO1) is reduced by *dcr-1* knockdown. These data illustrate the challenge in fully depleting the capacity for miRNA biogenesis using RNAi strategies, which in any case has associated consequences for cell survival.

Nevertheless, by focusing in on the non-miRNA reads, we do observe that the contribution of these species was about twice as large in the *dcr-1* library relative to the *gfp* library (Figure 7B, right graphs). For example, hpRNA-derived siRNAs, which are generated by Dcr-2, increased by 2.75-fold in the *dcr-1* library (Supplementary Table S2). Therefore, there is increased promiscuity in substrate acceptance by AGO1 upon depletion of Dcr-1. This interpretation was bolstered by quantification of mature miR-451 reads, which increased 8.34-fold from the *gfp* dsRNA library to the *dcr-1* dsRNA library (from 1772 reads/56.1 RPM to 17 829 reads/468 RPM).

With this in mind, we rescanned the genome with these AGO1-IP data in search of reads that mapped to short hairpins that furthermore (i) yielded dominant 5 p species exhibiting 3′ heterogeneity, (ii) generated longer reads that extend across the terminal loop and (iii) exhibited enhanced accumulation in *dcr-1* knockdown relative to *gfp* knockdown conditions. This analysis revealed some potential candidates. The locus depicted in Figure 7C was enriched 9.13-fold upon *dcr-1* knockdown and generated typical 21–22 nt miRNA-sized reads that crossed the terminal loop. As well, we observed 28 nt reads that shared the 5′ end of the shorter dominant reads, and these extended down the 3′ arm to a position that was reminiscent of a potential hairpin cleavage product. Although the hairpin pairing is not perfect, the unpaired nucleotides are in positions analogous to ones that are compatible with miR-451 biogenesis (11). Another locus with similar structural properties and *dcr-1* enrichment is shown in Supplementary Figure S5. We recognize that these loci are not very abundantly expressed, with the former locus reaching 1.37 RPM and the latter reaching 1.44 RPM in *dcr-1* AGO1-IP libraries. On the other hand, these experiments provide plausibility for the scenario that the miR-451 biogenesis tests we conducted may reflect the potential existence of endogenous Dicer-independent substrates in *Drosophila*.

## DISCUSSION

### Ago-mediated miRNA biogenesis in invertebrate species

**Figure 8.**
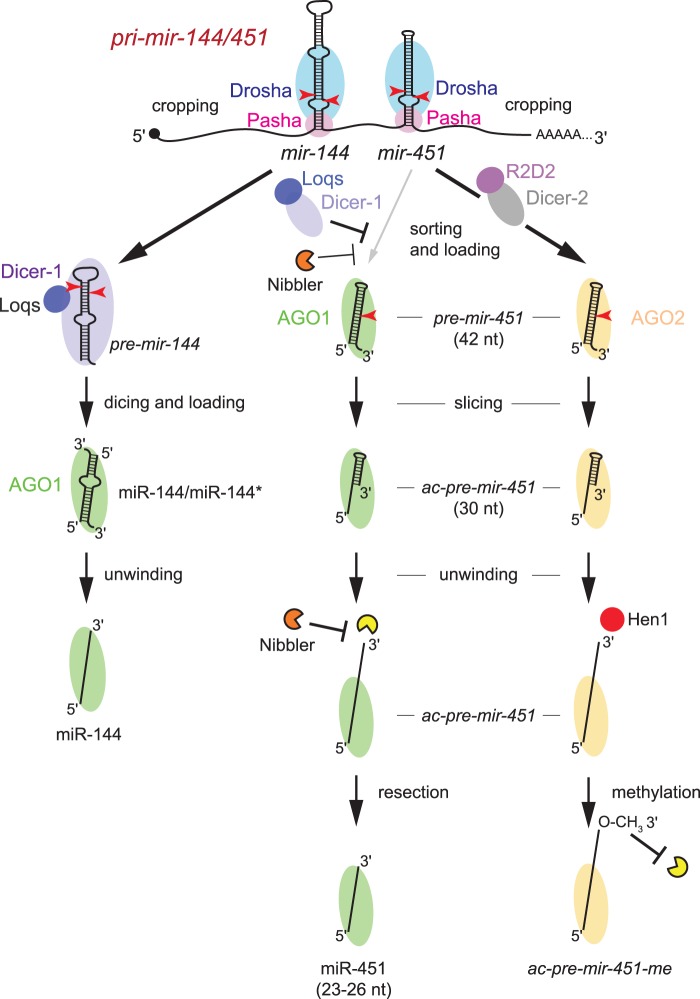
Model for the intertwined pathways that handle the processing of *mir-451*-type substrates in *Drosophila*. The primary hairpins for *mir-144* and *mir-451* are both cropped by the nuclear Microprocessor, composed of the RNase III enzyme Drosha and its dsRBD partner Pasha. Canonical *pre-mir-144* is a substrate for the cytoplasmic dicing complex, composed of the RNase III enzyme Dicer-1 and its dsRBD partner Loqs. This generates a miRNA/star duplex, which is loaded into AGO1, which releases the star strand to yield a mature single-stranded AGO1 complex. The *pre-mir-451* hairpin is too short to be diced, and can be directly loaded into either AGO1 or AGO2. However, due to competition from Dicer-1/Loqs-generated pre-miRNA hairpins, and its well-paired stem that resembles siRNA duplexes that are preferred by the RNAi loading machinery composed of the RNase III enzyme Dicer-2 and its dsRBD partner R2D2, bulk pre-mir-451 associates with AGO2. Those *mir-451* hairpins that load into AGO1 are sliced on the 3′ arm to yield 30 nt *ac-pre-mir-451*, whose 3′ end is resected by a soluble, EDTA-sensitive 3′–5′ exoribonuclease to yield mature miR-451. This process is in competition with the 3′–5′ exoribonuclease Nibbler, which can shorten the 3′ ends of long miRNAs in AGO1 complexes. Those mir-451 hairpins that load into AGO2 are cleaved on the 3′ arm, yielding *ac-pre-mir-451* species that are modified at their 3′ termini by the Hen1 methyltransferase, which prevents any further 3′ resection.

Bulk miRNAs in plants and animals are produced by canonical pathways, consisting of a series of seemingly essential biogenesis steps (3). However, such rules have proven made to be broken, and the recognition of diverse non-canonical strategies for miRNA biogenesis indicates a great deal of flexibility in the possibilities for the processing and metabolism of short RNA molecules (6). Notably, it has sometimes proven possible to import a completely foreign small RNA system into a ‘naïve’ host and have it be functional. For example, the human RNAi machinery is functional when introduced into *Saccharomyces cerevisiae* (46), and the bacterial CRISPR-Cas9 system has now been exploited in diverse eukaryotic systems (47). In this study, we imported the vertebrate Dicer-independent, Ago-dependent *mir-451* system into *Drosophila* cells and animals, and found it to be indeed functional. The maturation of *mir-451*-class substrates in vertebrate Ago2 and fly AGO1 appears similar. However, the segregation of the *Drosophila* miRNA and siRNA pathways introduces some unexpected twists, since *mir-451*-type hairpins are preferentially routed to fly AGO2 (Figure 8). In this effector, the biogenesis of *ac-pre-mir-451* is arrested, due to methylation of its 3′ end by Hen1.

The *mir-451* system is not the only Dicer-independent pathway known. Several species of budding yeast, such as *Saccharomyces castellii* and *Candida albicans*, utilize non-canonical ‘Dicer’ proteins to generate small RNAs that load Ago (48). These species use RNase III proteins that are not evolutionarily related to canonical Dicer proteins, and they utilize a distinct mechanism to measure cleavage sites for small RNA duplexes (49). In addition, *Schizosaccharomyces pombe* is capable of loading Dicer-independent ‘primal RNAs’ directly into Ago1 (50). These yeast pathways differ from *mir-451*-type substrates in that they do not generate specific miRNAs, but instead sample short RNAs from across transposons, or from the transcriptome in general. However, *Neurospora crassa* can utilize ribonucleases other than Dicer, such as the mitochondrial RNase MRPL3, to generate specific small RNAs that load into its Argonaute proteins (51). In addition, *Neurospora* Argonaute QDE-2 serves essential non-catalytic functions for the biogenesis of certain non-canonical miRNAs (52). Most recently, we used a ‘mix-and-match’ approach to combine Drosha-independent viral miRNA biogenesis strategies with Dicer-independent *mir-451* biogenesis, yielding multiple types of RNase III-independent miRNA substrates in mammalian cells (53). Studies such as these suggest that there may yet be additional endogenous Dicer-independent miRNA pathways still to be recognized.

Another theme that grows from the *mir-451* system is the appreciation that Ago proteins are not only receptacles of processed short RNAs, but can play important roles in their biogenesis. For example, it was recently found that the Slicer activity of *C. elegans* ALG1/2 is required, in some still undefined role, to promote miRNA biogenesis (54). Altogether, such findings may be potentially interpreted to reflect a precedent ‘Ago-first’ small RNA world that may have existed before the molecular coupling of RNase III enzymes with Ago proteins that is currently deeply embedded in most species with the capacity for RNAi. Our initial sequencing analyses of *dcr-1* knockdown in S2 cells indicate that this condition permits non-canonical substrates to accumulate in AGO1 complexes (Figure 7). Therefore, it should be interesting in the future to sequence AGO1-IP contents from *dcr-1* depletions of other types of cultured cells, or perhaps by examining *dcr-1* mutant tissues. Such profilings might reveal endogenous Ago-dependent substrates that are not predominantly expressed in S2 cells.

### Unexpected interplay of 3′–5′exoribonucleases for maturation of ac-pre-miRNAs

Although the identity of the 3′ resectase that matures miR-451 is presently elusive, our studies provide useful information regarding this process. First, our finding that the maturation of *ac-pre-mir-451* in AGO2 is blocked in a Hen1-dependent manner, provides strong evidence that 3′ resection is performed by a 3′–5′ exoribonuclease. Moreover, these data indicate that fully matured miR-451 species do not exchange between *Drosophila* AGO1 and AGO2 complexes, similar to our previous findings that mature miR-451 species do not exchange between mammalian Ago2 and non-slicing Argonautes such as Ago1 and Ago3 (11).

Second, we used knockdown assays to rule out that the Nibbler 3′–5′ exoribonuclease, which trims mature single-stranded miRNAs in AGO1 (30,31), is required for maturation of miR-451. It is currently difficult to imagine how Nibbler distinguishes these classes of ‘long’ RNAs associated with AGO1, and indicates mechanistic sophistication by this enzyme that remains to be understood. Beyond this, we unexpectedly observed that depletion of Nibbler actually increased the 3′ resection of miR-451. Taken at face value, these data suggest a competition for substrate access between Nibbler and the ‘Resector’ enzyme for miR-451-type molecules, and conversely, the existence of preferred substrates of these exoribonucleases (Figure 8). Since *nibbler* knockdown also seemed to increase the total amount of *mir-451*-associated species in AGO1 (Figure 6), it also seems that Nibbler may also regulate the flux of loading or perhaps turnover of AGO1 complexes. Either outcome would represent a novel function for Nibbler, which has thus far been implicated only in the trimming of species in mature AGO1 complexes (30,31). These scenarios deserve further study.

Third, we used an *in vitro* assay to show that miR-451 resectase activity resides in a soluble fraction and is likely Mg sensitive. Curiously, we were barely able to detect ‘Resector’ activity from S2 cell lysates, but it was more robust in ovary lysates. This is notable, since a conceptually similar activity in silkworm ovarian cells trims the 3′ ends of piRNAs in Piwi proteins (‘Trimmer’), but is apparently completely insoluble (42). To our knowledge, Trimmer activity has not been investigated in *Drosophila*, but these observations suggest that ovarian cells may harbor distinct 3′–5′ exoribonucleases, with specificity for immature RNAs loaded into Piwi proteins and immature RNAs loaded into Slicer Argonaute proteins. Such a conjecture awaits knowledge of the cognate molecular identities of these enzymes. Along with our previous finding that the RNA exosome trims the 3′ ends of a subclass of splicing-derived hairpins that require trimming to generate the pre-miRNA substrate (40), and the recent report that the Perlman syndrome 3′–5′ exoribonuclease Dis3L2 specifically degrades uridylated *pre-let-7* hairpins (55), there is clearly a diversity of exoribonucleases that shape the 3′ ends of small RNAs relevant to Argonaute pathways (56). It will be productive in the future to understand the full range of RNases that regulate small RNA sequence, abundance, and function, as well as to understand the nature of their potentially competitive or collaborative activities.

## ACCESSION NUMBERS

The sRNA sequencing data were deposited at the NCBI Gene Expression Omnibus under accession GSE51585.

## SUPPLEMENTARY DATA

Supplementary Data are available at NAR Online.

## FUNDING

Work in E.C.L.’s group was supported by the Burroughs Wellcome Foundation and [R01-GM083300]; Fellowship from the Swedish Society for Medical Research (to J.O.W.). Funding for open access charge: NIH [R01-GM083300].

*Conflict of interest statement*. None declared.

## Supplementary Material

Supplementary Data
