# Supplementary material for: Intertwined pathways for Argonaute-mediated microRNA biogenesis in Drosophila
Source: Nucleic Acids Res. 2013 Nov 12;42(3):1987–2002. doi: 10.1093/nar/gkt1038 (PMC3919586; doi:10.1093/nar/gkt1038)
Supplement: Supplementary Data [file supp_42_3_1987__index.html]

Intertwined pathways for Argonaute-mediated microRNA biogenesis in Drosophila — Intertwined pathways for Argonaute-mediated microRNA biogenesis in Drosophila — Supplementary Data 

# Intertwined pathways for Argonaute-mediated microRNA biogenesis in *Drosophila*

## Supplementary Data

files

**Files in this Data Supplement:**

- Supplementary Data - pdf file
