# Supplementary material for: Intertwined pathways for Argonaute-mediated microRNA biogenesis in Drosophila
Source: Nucleic Acids Res. 2013 Nov 12;42(3):1987–2002. doi: 10.1093/nar/gkt1038 (PMC3919586; doi:10.1093/nar/gkt1038)
Supplement: Supplementary Data [file supp_gkt1038_nar-02496-y-2013-File010.pdf]

Yang, et al

Intertwined pathways for Argonaute-mediated microRNA biogenesis in *Drosophila*

SUPPLEMENTARY FIGURE LEGENDS

Supplementary Figure 1. 3' modification of *ac-pre-mir-451* species in AGO2. We immunoprecipitated AGO1 and Flag-AGO2 complexes from S2R+ cells transfected with a *mir-144/451* construct. The ~30nt *ac-pre-mir-451* is predominantly associated with AGO2 complexes, and a minor population of trimmed species are associated with AGO1 complexes. The bulk ~30nt species in the input, as well as in AGO2-IP, were largely resistant to ß-elimination, while the miR-451 contents of AGO1 were degraded.

Supplementary Figure 2. Knockdown screen for miR-451 resecting factors. We co-depleted various annotated ribonucleases and Dcr-1 from S2R+ cells stably transformed with *MT-DsRed-mir-144/451*. Following incubation with dsRNA, we induced miRNA expression and assessed the resultant small RNAs using Northern blotting. The efficacy of Dcr-1 knockdown was shown by the lack of most mature miR-144 and miR-34 species, and accumulation of their pre-miRNAs. However, none of these knockdowns preferentially stabilized 30nt *ac-pre-mir-451*. Knockdown of CG9247 increased the amount of 3' trimmed miR-451 (compare to adjacent *gfp* knockdown lane). This RNase was recently renamed Nibbler, and shown to trim "long" miRNAs in AGO1 complexes. Consistent with this, cells depleted of CG9247 accumulated the longest mature miR-34 species (keeping in mind that overall signals for mature miR-34 are weak, due to codepletion of Dcr-1).

Supplementary Figure 3. Depletion of Dcr-1 and Nibbler enhances 3' trimming of miR-451. We treated S2R+ cells stably transformed with *MT-DsRed-mir-144/451* with dsRNA against *gfp*, *dcr-1*, and/or *nibbler*, then induced the miRNA construct by adding copper to the media, and assessed small RNAs using Northern blotting. Control *gfp* knockdown cells accumulate very little 3' trimmed miR-451. In contrast, *dcr-1* knockdown cells accumulate substantial 3' trimmed miR-451 species, and this can be enhanced by co-depletion of Nibbler. In addition, depletion of Nibbler alone can increase the accumulation of 3' trimmed miR-451. The efficacy of Dcr-1 knockdown is evidenced by

the loss of mature miR-144-3p and miR-34, and the efficacy of Nibbler knockdown is evidenced by the accumulation of untrimmed miR-34 species.

Supplementary Figure 4. Preliminary in vitro processing assays for the miR-451 3' resectase. (A) We purified AGO1 from S2 cells treated with *GFP* dsRNA, loaded it with 5' radiolabeled 42 nt *pre-mir-451* hairpin, and then treated it with the indicated lysates. No processed products were observed. (B) Control incubation shows that 5' radiolabeled *pre-mir-451* is retained on Myc-beads even after extensive washing, which likely obscured any *pre-mir-451* genuinely loaded into AGO1.

Supplementary Figure 5. Candidate short hairpin whose associated small RNAs cross the predicted terminal loop and were enriched in an AGO1-IP library from S2R+ cells treated with dsRNA against *dcr-1*, relative to cells treated with *gfp* dsRNA.

Supplementary Table 1. Primer sequences for miRNA expression constructs, miRNA sensors, dsRNA amplicons, and Northern probes.

Supplementary Table 2. Statistics of AGO1-IP libraries from S2R+ cells treated with dsRNA against *gfp* or *dcr-1*.

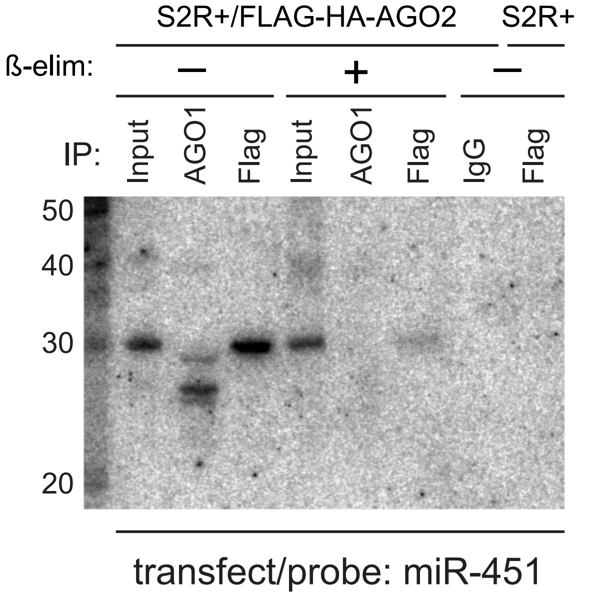

Yang et al
Supplementary Figure 1

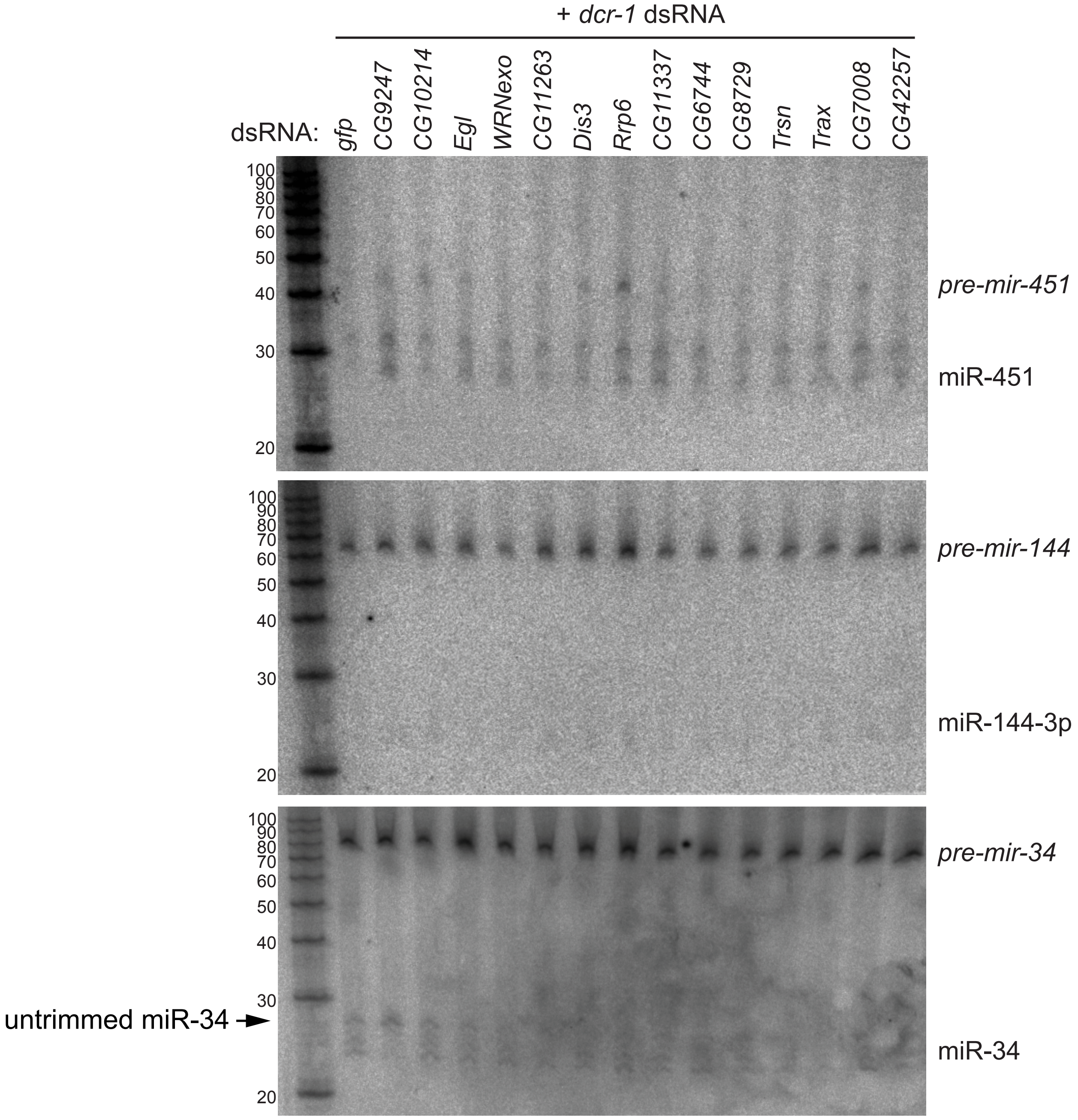

Yang et al
Supplementary Figure 2

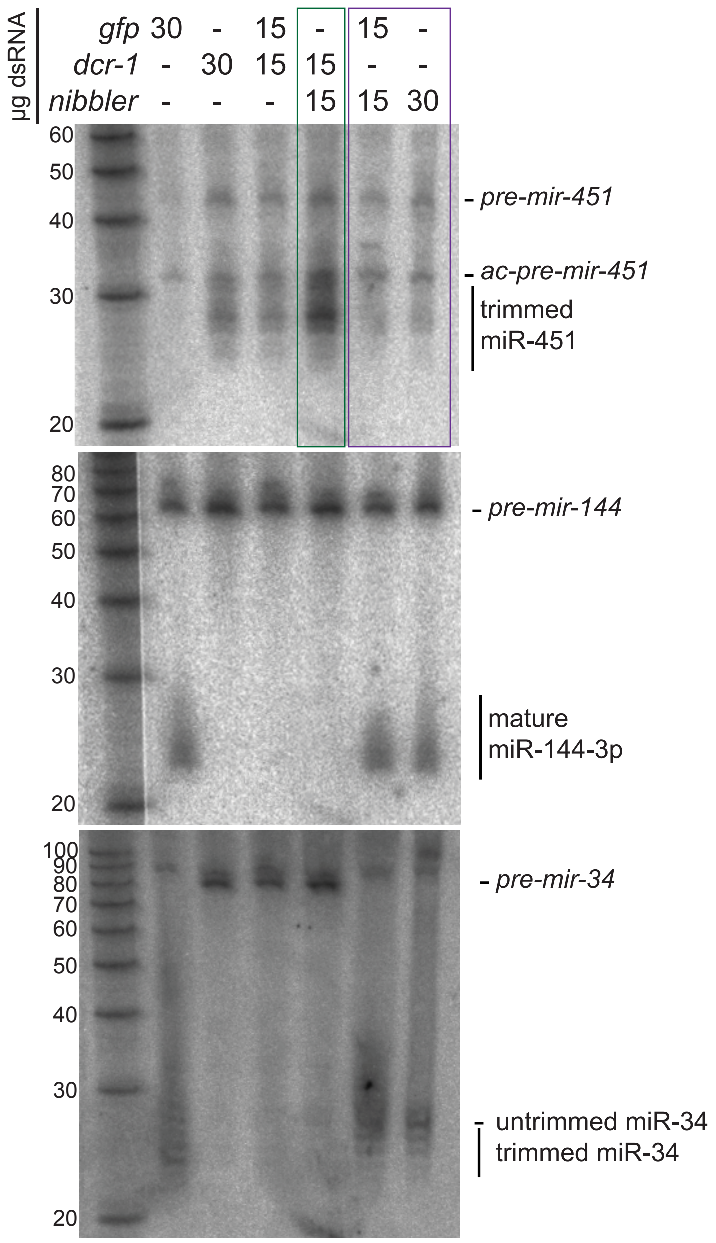

Yang et al
Supplementary Figure 3

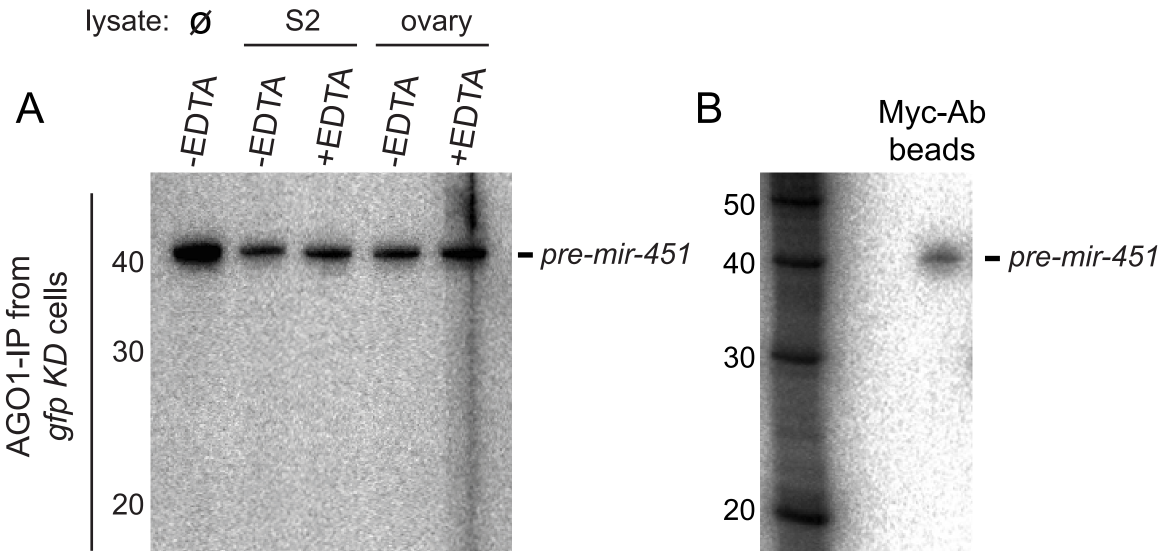

Yang et al
Supplementary Figure 4

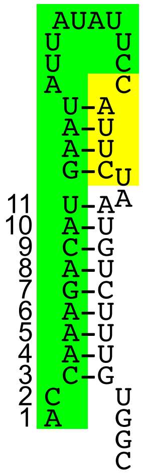

21.7x enrichment (normalized over all hairpin reads)

chr3R:19684934-19685054(-)	read length	ds-gfp AGO1-IP	ds-dcr-1 AGO1-IP
ATTATTGGCAACCAAAGACATGAATATTATATTCCATTCTAATGTCTTTGTGGCAACCGTGT			
....(((.((..(((((((((((((..........))))..))))))))))).)))......			
..........ACCAAAGACATGAATATTATATTC............................	24	0	8
..........ACCAAAGACATGAATATTATATTCCATTCT......................	30	0	11
..........ACCAAAGACATGAATATTATATTCCAT.........................	27	0	4
..........ACCAAAGACATGAATATTATATTCC...........................	25	1	2
..........ACCAAAGACATGAATATTATATTCCATT........................	28	0	6
..........ACCAAAGACATGAATATTATATT.............................	23	0	7
...........CCAAAGACATGAATATTATATTCCA..........................	25	0	6
(singleton reads not shown)			

Yang et al

Supplementary Figure 5

Supplementary Table 1. Primer sequences

dsRNA primers

CG9247_F	CGACGAGTGCCTTATCATTG	536 bp	Nibbler, DnaQ-like-exo
CG9247_R	TGGCTGCATAAAGAATCTGC		
CG7008_F	CCGGCGATACGGTAGTTATT	454 bp	Tudor-SN
CG7008_R	GGATTCTCATGGACCACTT		
CG11337_F	TTCGCGAACTGATCTTTGAG	541 bp	Rrp41, Rnase PH, 3'5'exo; RNA binding; ribonucleotransferase
CG11337_R	TCCTGCAGGTGCTTAGTGTC		
CG10214_F	TCGGATTGTCCCTGAACC	405 bp	DnaQ-like-exo
CG10214_R	GGTCGGTGTAGACGGAGTTC		
CG42257_F	TCCAGCAGAAGACTGTGGAC	454 bp	Also known as CG6393, Snp; control, RRM domain PABP
CG42257_R	ACGAACCTGGGATTAAGCTG		
Dis3_F	ATCCTTATCCAAGGACGCGAGTC	504 bp	
Dis3_R	CTCCAGCAGGATAACCTCGTTCT		
Rrp6_F	TGATCCTACGCGATGATATGCATA	516 bp	Exosc10
Rrp6_R	ACTCGAAGATTCCGCGAAGTG		
egl_F	CCTGAGCTCATTCCTCAAGC	490 bp	CG4051, DnaQ-like exo, egl-like
egl_R	GGTGTTCGCACTGGGATAGT		
CG11263_F	CTCCCGGAAAACATTCGTTA	479 bp	DnaQ-like exo, no intron!
CG11263_R	CAGCCTCCAGTCACCTTCTC		
Trsn_F	CAGTCTGAGGGCTTCCATCT	361 bp	
Trsn_R	TTTCATTTTAATAAACGCCTTATCC		
Trax_F	CAAGCTGAGCCGTGACATTA	431 bp	
Trax_R	TGGGGTCTACGAAAAACTGG		
CG6744_F	TTCCGACTGTGCCATATGAA	457 bp	3'5'exo;RNase H-like. ubiquitous
CG6744_R	TGAGTGTGAAGCCTTTCGTG		
WRNexo_F	TCCAGATATGCGTGGATGAA	417 bp	3'5'exo;RNase H-like. ovary specific.
WRNexo_R	GATGGCGGCGTACATTAGTT		
CG8729_F	TGCCACATATGTTTCGATAGAC	379 bp	rnh1
CG8729_R	CAGATCATCCTCGGCTAGGT		

qPCR primers

qCG9247_F	CCTTGGCAAGAAACTGAACA	
qCG9247_R	ATCGATGGCTGCATAAAGAA	
qCG7008_F	TGAAGACAGGCACCGATAAA	
qCG7008_R	TTCCACCACTGTTCCAGAGA	
qCG11337_F	TCAACTATCGCCTCAAATGC	
qCG11337_R	GGTCATGTTACGTCCATTGC	
qCG10214_F	ATGTGTCCACCATCAAGGAA	no intron, may amplify genomic DNA
qCG10214_R	TTGATGCTTTCTCGGATGTC	
qCG42257_F	GATAATTGAATTCCCAGCAGTG	might amplify 2 fragment
qCG42257_R	CCACAGTCTTCTGCTGGATG	
qEgl_F	TGACGTTGCTCTTCTTCCTG	
qEgl_R	TCTTCAGACCCGACTGACTG	
qWRNexo_F	ATCCAGATATGCGTGGATGA	
qWRNexo_R	GCTTTCGGAAATCGTTCTTT	
qCG6744_F	CAGCTAAGCAACATCTCGGA	
qCG6744_R	GATTGGACTCGTTTGAGCTG	
qCG8729_F	GGTGCCTTCCAGTTTGAAAT	
qCG8729_R	TGCGTTTAGCTGGTGATTCT	
qRrp6_F	ATGCCGCGAACCCCGAAAA	
qRrp6_R	GAATGCCTCAACATCTTCTGAAGCGG	
qDis3_F	ATGCAAACTTTACGCGAATTTACGCG	
qDis3_R	TTGGAAACACTCCCGGCACA	

Northern probes

miR-451-5p	AACTCAGTAATGGTAACGGTTT
miR-144-3p	AGTACATCATCTATACTGTA
miR-199a-3p	TAACCAATGTGCAGACTACTGT
miR-19a-3p	TCAGTTTTGCATAGATTTGCACA
2S rRNA	TACAACCCTCAACCATATGTAGTCCAAGCA
Bantam	CAGCTTTCAAAATGATCTCACT
miR-34-5p (Exiqon LNA)	CACAACCAGCTAACCACACTGCCA
miR-277 (Exiqon LNA)	TGTCGTACCAGATAGTGCATTTA
hp-CG4068B (Exiqon LNA)	GGAGCGAACTTGTTGGAGTCAA

miRNA expression constructs

UAS-DsRed-hsa-mir144/451_F	ataagaatgcggccgcTAATGGGGCCCTGGCTGGG
UAS-DsRed-hsa-mir144/451_R	ccgctcgagTAACAAGAAATCCTCCTGCCTTG

miRNA sensors (to make 2x sensors in psiCHECK vector)

mir-451-5p2si_A	GGCCGCcAACTCAGTAATGGTAACGGTTTtcaacaatcaccAACTCAGTAATGGTAACGGTTTtG
mir-451-5p2si_B	TCGACaAAACCGTTACCATTACTGAGTTgtgatttgttgaAAACCGTTACCATTACTGAGTTgGC
mir-199-3p2si_A	GGCCGCcAACCAATGTGCAGACTACTGTAtcaacaatcaccAACCAATGTGCAGACTACTGTAtG
mir-199-3p2si_B	TCGACaTACAGTAGTCTGCACATTGGTTgtgatttgttgaTACAGTAGTCTGCACATTGGTTgGC
mir-19a-3p2si_A	GGCCGCcTCAGTTTTGCATAGATTTGCACAtcaacaatcaccTCAGTTTTGCATAGATTTGCACAtG
mir-19a-3p2si_B	TCGACaTGTGCAAATCTATGCAAAACTGAggtgattgttgaTGTGCAAATCTATGCAAAACTGAgGC

miRNA sensors (to make 4x sensors in tubulin-GFP vector)

mir-451-5p_2si_A_SalI	tcgacaaaaAACTCAGTAATGGTAACGGTTTaaaactcgacaaaaAACTCAGTAATGGTAACGGTTTaaaac
mir-451-5p_2si_B_XhoI	tcgagttttAAACCGTTACCATTACTGAGTTttttgtcgagttttAAACCGTTACCATTACTGAGTTtttg
mir-144-3p_2si_A_SalI	tcgacaaaaAGTACATCATCTATACTGTAaaaactcgacaaaaAGTACATCATCTATACTGTAaaaac
mir-144-3p_2si_B_XhoI	tcgagttttTACAGTATAGATGATGTACTttttgtcgagttttTACAGTATAGATGATGTACTttttg

transfer 144/199RP and 144/19aRP constructs from pcDNA6.2 to pUAST dsred (cold fusion)

p6.2xhotopUAST_R	attatgtCACACCACAGAAGTAAGGctcgagCCCATTATTAAGGGCGAATT
p6.2xhotopUAST_F2	CAGCTGCCCGGCTACTACTACGTGGActcgagGTACAAAAAAGCAGGCTCCG

Supplementary Table 2. Small RNA library statistics.

Library	M035	M036	
**Description**	**S2-R+ cells, Ago1-IP, dsGFP**	**S2-R+ cells, Ago1-IP, dsDcr1**	
**nr reads**	38,540,339	50,200,101	
**trimmed**	37,897,867	49,435,406	
**size <= 15 nt**	36,845,419	45,814,495	
**mapped dm3 + vector**	31,581,148	38,072,284	
**mapped to spike ins**	106,549	181,984	
**miRNAs**	30,669,745	35,674,210	
**hpRNAs**	113,846	377,131	
**cis-natRNAs**	3,170	7,933	
**rRNAs**	445,127	914,943	
**tRNAs**	23,547	134,731	read nrs
**snoRNAs**	4,896	8,800	
**snRNAs**	4,294	5,392	
**Transposable elements**	149,556	454,268	
**Other**	166,967	494,876	
**miRNAs**	97.114%	93.701%	
**hpRNAs**	0.360%	0.991%	
**cis-natRNAs**	0.010%	0.021%	
**rRNAs**	1.409%	2.403%	normalized to library size
**tRNAs**	0.075%	0.354%	
**snoRNAs**	0.016%	0.023%	
**snRNAs**	0.014%	0.014%	
**Transposable elements**	0.395%	0.919%	
**Other**	0.529%	1.300%	
**miRNAs**		0.22	
**hpRNAs**		**1.73**	
**cis-natRNAs**		**1.32**	
**rRNAs**		**1.04**	
**tRNAs**		**2.52**	normalized to spike ins
**snoRNAs**		0.85	
**snRNAs**		0.33	
**Transposable elements**		**1.60**	
**Other**		**1.57**	
**miRNAs**		-0.05	
**hpRNAs**		**1.46**	
**cis-natRNAs**		**1.05**	log ratio vs dfGFP (normalized for lib size)
**rRNAs**		0.77	
**tRNAs**		**2.25**	
**snoRNAs**		0.58	
**snRNAs**		0.06	
**Transposable elements**		**1.33**	
**Other**		**1.30**	

spike-ins

**name**	**M035**	**M036**	**Sequence**
hsamiR-629	22,353.0	32,410.0	GTTCTCCCAACGTAAGCCCAGC
mir-877-5p	17,878.0	45,315.0	GTAGAGGAGATGGCGCAGGG
gga-wnt3-5p	15,798.0	19,783.0	TTGGGAAGGAACAAAGCATGACTT
131741-3p	11,920.0	24,276.0	TCACCAGCCCTGTGTTCCCTAG
98785	8,811.0	34,777.0	CCTCACCATCCCTTCTGCCTGCAG
GL2_target	9,706.0	8,016.0	ACGTACGCGGAATACTTCGATT
hsa-miR-142-5p	8,259.0	8,008.0	CATAAAGTAGAAAGCACTACT
GFP_target	4,412.0	3,707.0	AGCAAGCTGACCCTGAAGTTCAT
gga-ADAMTS10-5p	4,466.0	3,705.0	TTGGGGACACCATCAGAACAGCCA
hsa-miR-142-3p	2,946.0	1,987.0	TGTAGTGTTTCCTACTTTATGGA
